# Arabidopsis CRL4 Complexes: Surveying Chromatin States and Gene Expression

**DOI:** 10.3389/fpls.2019.01095

**Published:** 2019-09-17

**Authors:** Sandra Fonseca, Vicente Rubio

**Affiliations:** Department of Plant Molecular Genetics, Centro Nacional de Biotecnología (CNB-CSIC), Madrid, Spain

**Keywords:** CRL4, DWD, DCAF, protein degradation, chromatin, transcriptional regulation, ribosome, RNA export

## Abstract

CULLIN4 (CUL4) RING ligase (CRL4) complexes contain a CUL4 scaffold protein, associated to RBX1 and to DDB1 proteins and have traditionally been associated to protein degradation events. Through DDB1, these complexes can associate with numerous DCAF proteins, which directly interact with specific targets promoting their ubiquitination and subsequent degradation by the proteasome. A characteristic feature of the majority of DCAF proteins that associate with DDB1 is the presence of the DWD motif. DWD-containing proteins sum up to 85 in the plant model species Arabidopsis. In the last decade, numerous Arabidopsis DWD proteins have been studied and their molecular functions uncovered. Independently of whether their association with CRL4 has been confirmed or not, DWD proteins are often found as components of additional multimeric protein complexes that play key roles in essential nuclear events. For most of them, the significance of their complex partnership is still unexplored. Here, we summarize recent findings involving both confirmed and putative CRL4-associated DCAF proteins in regulating nuclei architecture remodelling, DNA damage repair, histone post-translational modification, mRNA processing and export, and ribosome biogenesis, that definitely have an impact in gene expression and *de novo* protein synthesis. We hypothesized that, by maintaining accurate levels of regulatory proteins through targeted degradation and transcriptional control, CRL4 complexes help to surveil nuclear processes essential for plant development and survival.

## Introduction

Proteins participate in every function of a living cell, from metabolic to signalling events, and as cellular structural components. Protein synthesis and degradation are tightly regulated mechanisms that enable precise control of the abundance and function of each particular protein. In the nucleus, the activities of a myriad of proteins converge to maintain and shape chromatin structure and function, which determine gene expression and accumulation of their protein products depending on the cellular and environmental conditions. Chromatin is highly organized and packed into nucleosomes, a compact and stable structure capable of inhibiting transcription, DNA replication, and repair. The nucleosome core is composed of a histone protein octamer around which is wrapped ∼147 bp of DNA (Luger et al., 1997). Histone post-translational modifications might relax or compact this structure allowing a more efficient progression of polymerases, regulating transcription output and *de novo* protein synthesis. Concomitantly, tight coordination of transcription with RNA processing and export mechanisms is required to ensure the fine-tuning of cellular protein homeostasis. This requires not only protein synthesis but also refined and highly precise degradation mechanisms. Proteasome-mediated degradation is one of the best-studied mechanisms to terminate protein life cycle and is based in the targeted degradation of ubiquitin-marked proteins. The ubiquitin (Ub) mark is selectively conjugated to protein targets by a conserved enzymatic cascade in which E3 Ub ligases provide the substrate specificity. CULLIN RING ligases (CRLs), the largest class of E3s, represent a family of modular complexes, consisting in eukaryotes of at least seven different cullin scaffold proteins, each of them serving as a building block for the assembly of tens or more multi-subunit CRLs (Deshaies and Joazeiro, 2009). Among this class, CULLIN4 (CUL4) RING ligases (CRL4) control key aspects of cell biology in eukaryotes, including cell cycle progression and DNA damage repair and replication (Jackson and Xiong, 2009; Biedermann and Hellmann, 2011). CUL4 binds RING finger protein (ROC1/RBX1) to recruit the E2 Ub-conjugating enzyme and to DAMAGED DNA BINDING PROTEIN1 (DDB1). The latter functions as a recruiter protein that interacts with DDB1, CUL4-ASSOCIATED FACTORS (DCAF) substrate adapters, which recognize specific proteins to target them for ubiquitination and subsequent proteasomal degradation. DCAFs are therefore fundamental to confer substrate specificity to CRL4 complexes (Hu et al., 2004; Bernhardt et al., 2006; Lee et al., 2008).

The observation that, from yeast to animals and plants, CRL4 conserves the capacity to interact with WD40-repeat proteins and the finding that each of these complexes generates functional E3 ligases led to the idea that WD40-repeat containing proteins work as DCAFs (Angers et al., 2006; He et al., 2006). The WD40 repeat spans 40–60 amino acids and is notable for a tryptophan-aspartic acid (WD) dipeptide at its C terminus, but exhibits only limited amino acid sequence conservation (Van Nocker and Ludwig, 2003; Higa and Zhang, 2007). The majority of DCAFs contain six or more WD40 motifs. Each WD40 repeat comprises a four-stranded antiparallel beta sheet that folds as a blade. WD40 blades are organized in a high order structure packed radially around a central axis to form a complete ß-propeller. This structural feature is common to all WD40 proteins whose structure has been determined (Zimmerman et al., 2010). Due to its intrinsic characteristics, the ß-propeller provides multiple surfaces making it prone to protein–protein interaction, and facilitating binding to diverse partners. In fact, WD40 proteins are often integral components of different multiprotein complexes (Zimmerman et al., 2010; Migliori et al., 2012).

DDB1 itself contains three distinct 7-blade ß-propellers (BP), designated BPA, BPB, and BPC (Li et al., 2006). Structural analysis showed that the BPB domain tethers DDB1 to CUL4, while BPA and BPC form a structurally coupled double propeller pointed away from the CUL4 scaffold, suggesting that DDB1 ß-propellers can generate three interaction surfaces that can work independently with different binding specificities. The intrinsic flexibility of this structure would allow accommodating diverse DCAFs with different shapes (Zimmerman et al., 2010). Noteworthy, in Arabidopsis, there are two genes encoding DDB1 proteins, DDB1A and DDB1B. Although their products are 91% identical they have partially distinct functions and may interact with different DCAF subsets to mediate ubiquitination of specific protein substrates in each case (Schroeder et al., 2002). Additionally, similar to the case of the human DDB1 interaction with the replication licensing factor CDT1, plant DDB1 proteins might also directly interact with certain protein substrates to trigger their destabilization (Hu et al., 2004; McCall et al., 2005).

Analysis of WD40 proteins that interact with DDB1 in animals revealed that a conserved motif within the WD40 repeats is required for this interaction—the DWD motif (also known as WDxR or DxR). This motif is a unique signature in proteins that provides a binding site for DDB1 and consists of 16 amino acids, from which 4 are highly conserved residues: Asp-7 (or Glu), Trp-13 (or Tyr), Asp-14 (or Glu), and Arg-16 (or Lys) (He et al., 2006; Lee et al., 2008).

**Figure 1:**
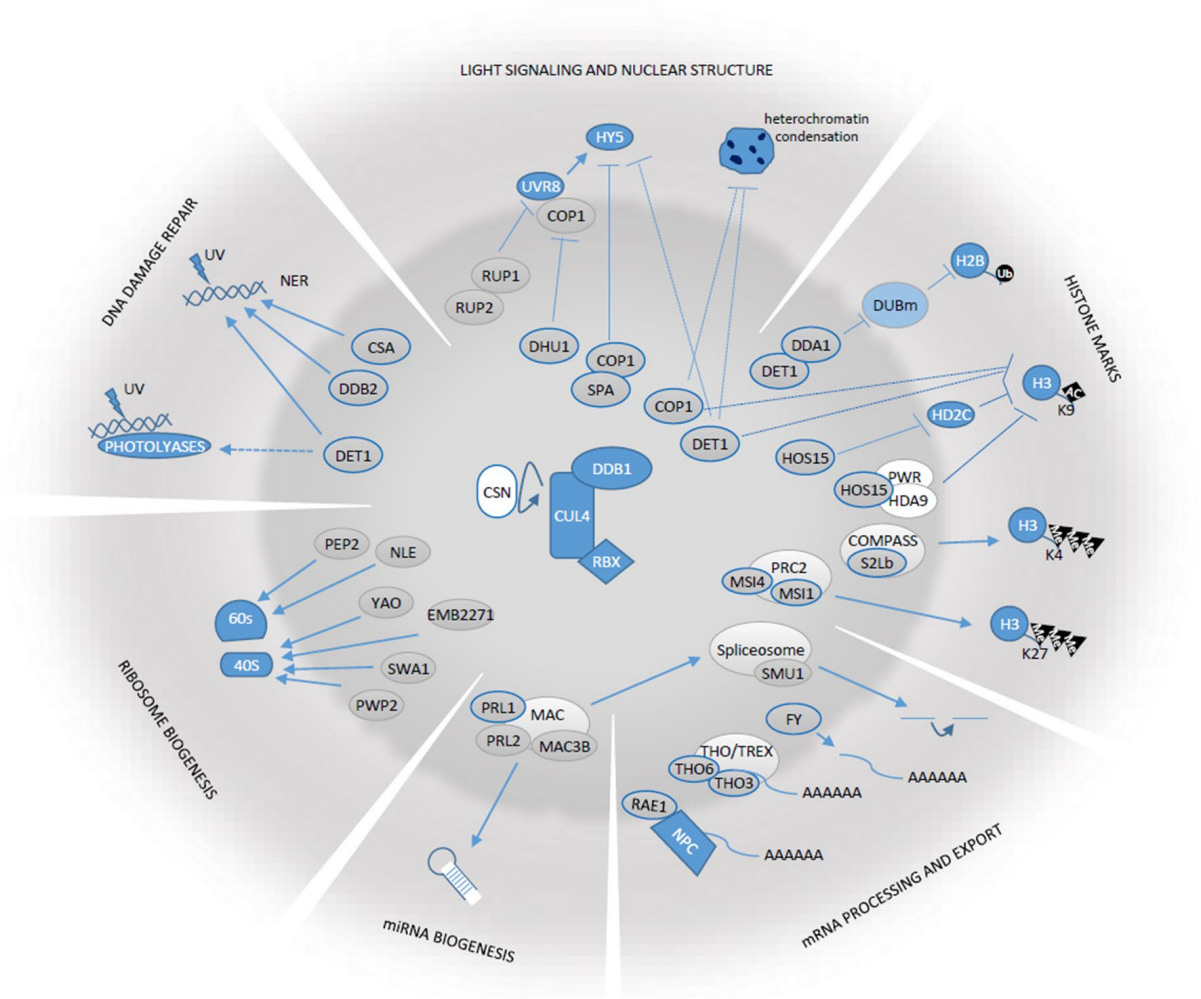
Schematic representation of the functions in which Arabidopis CDDD components and DWD-containing proteins play a role. Gray ovals in the central circle’s area correspond to CDDD and DWD proteins referred in this review. Those with thick blue borders correspond to proteins known to associate with CRL4 complexes. Arrows and blocked lines correspond to positive or negative regulatory relationships. Dashed lines indicate that the functional relationships are indirect and its molecular details are unknown.

*In silico* analysis has shown that the DWD protein family was in expansion during viridiplantae evolution (Tevatia and Oyler, 2018). Indeed, a large number of DWD proteins have been identified in Arabidopsis (85), rice (*Oryza sativa*; 78), and soybean (*Glycine max*; 161) (Lee et al., 2008; Bian et al., 2017). In the last decade, great advances have been made on the characterization of the molecular functions of DWD proteins as part of CRL4 and other multimeric complexes. From the 85 DWD Arabidopsis proteins described by Lee and co-workers in 2008, 53 have been at least partially characterized and 32 of those have an impact in chromatin homeostasis, transcriptional regulation, and *de novo* protein synthesis (**Table 1**; **Supplementary Table 1**). These proteins, together with the DDB1-associated CDDD module, involved in surveying chromatin states and gene expression as part of CRL4 complexes are the focus of this review (**Figure 1**).

**Table 1:** DCAF proteins characterized in Arabidopsis.

AGI	GENE NAME	DWD Present	Associates with DDB1	Molecular Function	Other interactors related with chromatin and/or gene expression	References
**NUCLEAR ARCHITECTURE AND LIGHT SIGNALLING**						
AT2G32950	COP1 (CONSTITUTIVE PHOTOMORPHOGENIC 1)	yes	yes	CRL4 E3 ligase activity; light signalling; photomorphogenesis; nuclear architecture; flowering; others.	COP1-SPA complex Targets TFs as HY5, HFR1, CO and others	Ang et al., 1998; Osterlund et al., 2000; Jang et al., 2005; Liu et al., 2008; Bourbousse et al., 2015
AT2G46340	SPA1 (SUPPRESSOR OF *PhyA* 1)	yes	yes	CRL4 E3 ligase activity; light signalling; photomorphogenesis; flowering	COP1-SPA complex	Hoecker et al., 1999; Saijo et al., 2003; Chen et al., 2010
AT4G11110	SPA2	yes	yes	Light signalling; photomorphogenesis; flowering	COP1-SPA complex	Laubinger and Hoecker, 2003; Laubinger et al., 2006; Chen et al., 2010
AT3G15354	SPA3	yes	yes	Light signalling; photomorphogenesis; flowering	COP1-SPA complex	Laubinger and Hoecker, 2003; Laubinger et al., 2006; Chen et al., 2010
AT1G53090	SPA4	yes	yes	Light signalling; photomorphogenesis; flowering	COP1-SPA complex	Laubinger and Hoecker, 2003; Laubinger et al., 2006; Chen et al., 2010
AT4G10180	DET1 (DE-ETIOLATED 1)	no	yes	Light signalling; photomorphogenesis; nuclear architecture; co-transcriptional repressor in circadian clock	CDDD complex; PIF1-4, LHY, HFR1 transcription factors; others	Pepper et al., 1994; Osterlund et al., 2000; Bernhardt et al., 2006; Lau et al., 2011; Dong et al., 2014; Bourbousse et al., 2015; Shi et al., 2015
AT3G13550	COP10 (CONSTITUTIVE PHOTOMORPHOGENIC 10)	no	yes	Ubiquitin ligase, enhancer of other ubiquitin ligases; photomorphogenesis	CDDD complex	Yanagawa et al., 2004; Lau and Deng, 2009
AT5G52250	RUP1 (REPRESSOR OF UVB-PHOTOMORPHOGENESIS 1)	yes	nd	CRL4 E3 ligase activity; UV-B signalling	UVR8	Gruber et al., 2010; Heijde and Ulm, 2013
AT5G23730	RUP2	yes	nd	CRL4 E3 ligase activity; UV-B signalling	UVR8	Gruber et al., 2010; Heijde and Ulm, 2013
AT4G34280	DHU1 (DWD HYPERSENSITIVE TO UV-B 1)	yes	yes	UV-B signalling	COP1, RUP1	Kim et al., 2014; Kim et al., 2017
**DNA DAMAGE REPAIR**						
AT1G27840	CSA-1 (COCKAYNE SYNDROME A)	yes	yes	CRL4 E3 ligase activity; nuclear excision repair; UV-B tolerance; genomic integrity	CSA heterotetramer	Zhang et al., 2010
AT5G58760	DDB2 (DNA BINDING PROTEIN 2)	yes	yes	CRL4 E3 ligase activity; nuclear excision repair; UV-B tolerance and genomic integrity	nd	Koga et al., 2006; Molinier et al., 2008
AT5G41560	DDA1 (DDB1-ASSOCIATED 1)	no	yes	H2B ubiquitination; ABA signalling	CDDD complex; Targets SGF11	Irigoyen et al., 2014; Nassrallah et al., 2018
AT4G10180	DET1	no	yes	DNA damage repair		Castells et al., 2010
**HISTONE AND DNA MARKS**						
AT5G58230	MSI1 (MULTICOPY SUPPRESSOR OF IRA1)	yes	yes	H3K27me3; parental imprinting; embryo development; cell cycle control	PRC2 (POLYCOMB REPRESSIVE COMPLEX 2)	Dumbliauskas et al., 2011
AT2G16780	MSI2	yes	nd	unknown	nd	Lee et al., 2008
AT4G35050	MSI3	yes	yes	unknown	nd	Pazhouhandeh et al., 2011
AT2G19520	MSI4		yes	H3K27me3; flowering time regulation	PRC2	Pazhouhandeh et al., 2011
AT4G10180	DET1	no	yes	H2B ubiquitination, Regulates H3K9 acetylation	Targets SGF11 and UBP22, part of the DUBm (deubiquitination module)	Benvenuto et al., 2002; Nassrallah et al., 2018; Guo et al., 2008
AT2G32950	COP1	yes	yes	Regulates H3K9 acetylation		Guo et al., 2008
AT5G14530	S2La (SWD2-LIKE A)	yes	nd	Flowering time regulation	nd	Fiorucci et al., 2019; Kapolas et al., 2016
AT5G66240	S2Lb	yes	yes	H3K4me3; cell wall thickening; anther dehiscence	COMPASS (COMPlex of Proteins Associated with Set1)	Beris et al., 2016; Fiorucci et al., 2019
AT5G67320	HOS15 (HIGH EXPRESSION OF OSMOTICALLY RESPONSIVE GENES 15)	yes	yes	CRL4 E3 ligase activity; regulates histone acetylation; cold tolerance and response to abiotic stresses	HD2C; CBFs; Conforms the HOS15-HDA9-PWR complex	Park et al., 2018a; Park et al., 2018b; Zhu D. et al., 2008; Suzuki et al., 2018; Mayer et al., 2019
**mRNA PROCESSING AND EXPORT**						
AT5G13480	FY (FLOWERING LOCUS Y)	yes	yes	Control of flowering; alternative polyadenylation; embryo development	FCA	Lee et al., 2008; Shi et al., 2009; Simpson et al., 2003
AT1G73720	SMU1 (SUPRESSOR OF MEC-8 AND UNC-52 1)	yes	nd	Alternative splicing	Spliceosome	Kanno et al., 2017
AT5G56130	TEX1/THO3 (TANSCRIPTION EXPORT 1)	yes	nd	mRNA export from nucleus; siRNA biosynthesis	TREX/THO complex	Lee et al., 2010; Yelina et al., 2010; Jauvion et al., 2010; Sørensen et al., 2017
AT2G19430	THO6	yes	yes	mRNA export from nucleus; siRNA biosynthesis; ABA signaling; Conforms a functional CRL4 E3 ligase	TREX/THO complex	Lee et al., 2010; Yelina et al., 2010; Jauvion et al., 2010; Sørensen et al., 2017
AT1G80670	RAE1 (RNA EXPORT FACTOR 1)	yes	yes	mRNA export; shuttling at the nuclear envelope	Nuclear pore complex	Lee et al., 2008; Pritchard et al., 1999; Okamura et al., 2015.
**miRNA BIOGENESIS**						
AT4G15900	PRL1 (PLEIOTROPIC REGULATORY LOCUS 1)	yes	yes	miRNA biogenesis; CRL4 E3 ubiquitin ligase activity; energy homeostasis; plant innate immunity	MAC complex (MOS4-Associated Complex); Targets AKIN10 kinase	Lee et al., 2008; Monaghan et al., 2009; Jia et al., 2017; Li et al., 2018; Wiborg et al., 2008
AT3G16650	PRL2	yes	nd	miRNA biogenesis	MAC complex	Jia et al., 2017; Li et al., 2018
AT2G33340	MAC3B	yes	nd	miRNA biogenesis; CRL4 E3 ubiquitin ligase activity; plant innate immunity	MAC complex	Jia et al., 2017; Li et al., 2018
**RIBOSOME BIOGENESIS**						
AT2G47990	SWA1 (SLOW WALKER 1)	yes	nd	18S rRNA processing; mitotic progression; megagametogenesis	nd	Shi et al., 2005
AT1G15440	PWP2 (PERIODIC TRYPTOPHAN PROTEIN 2)	yes	nd	18S rRNA processing; gametophyte and embryo sac development	nd	Missbach et al., 2013
AT4G05410	YAO (YAOZHE)	yes	no	18S rRNA processing; male and female gametogenesis; zygote development; cell division	nd	Li et al., 2010
AT4G21130	EMB2271	yes	nd	18S rRNA processing	nd	Li et al., 2010
AT5G15550	PEP2 (PESCADILLO ORTHOLOG 2)	yes	nd	28S and 5.8S rRNA processing; cell proliferation; cell cycle	PeBoW (PES, BOP, WDR12) complex	Zografidis et al., 2014; Ahn et al., 2016
AT5G52820	NLE (NOTCHLESS)	yes	nd	60 S ribosomal subunit maturation and assembly; cell growth and proliferation; female gametophyte development		Chantha and Matton, 2007; Chantha et al., 2010

The table summarizes protein function information within the scope of chromatin remodeling and additional nuclear events that affect gene expression. nd stands for non-determined.

## Nuclear Structure and Light as a Signal

Plants use light as an informational cue to control a multitude of physiological responses throughout their life cycle. Thus, light affects multiple aspects of plant development, including seed germination, seedling and leaf development, shade avoidance responses, and flowering (Jiao et al., 2007). Collectively, these responses are known as photomorphogenesis, which represents one of the best-studied developmental processes in plants (Kendrick and Kronenberg, 2012). Light perception and signalling are tightly regulated by components of the ubiquitin-proteasome system (the so-called COP/DET/FUS proteins), which modulate the stability and activity of both photoreceptors and transcriptional regulators (i.e., bZIP transcription factor ELONGATED HYPOCOTYL 5, HY5). In this process, DWD-containing CONSTITUTIVE PHOTOMORPHOGENIC1 (COP1) and SUPPRESSOR OF PHYA (SPA 1–4) proteins play a central role acting as part of CRL4 E3 Ub ligases that target a wide variety of photomorphogenesis promoting factors, including HY5, the photoreceptor PhyA, and the flowering time regulator CONSTANS (CO) in Arabidopsis (Lau and Deng, 2012).

COP/DET/FUS proteins include additional DCAF proteins that lack any WD40 repeats, but they associate to CRL4 complexes by physical binding to DDB1. In Arabidopsis, the most prominent representative is DE-ETIOLATED1 (DET1), a classic photomorphogenesis repressor (Pepper et al., 1994). In addition to DDB1, DET1 associates with COP10 and DET1, DDB1-ASSOCIATED1 (DDA1), both also non WD-40 proteins, to form a heterotetrameric CDDD module that binds to CUL4 (Schroeder et al., 2002; Yanagawa et al., 2004; Pick et al., 2007; Biedermann and Hellmann, 2011; Chen et al., 2006, Irigoyen et al., 2014). DET1 acts at different levels to repress photomorphogenesis during plant development. Thus, during seedling establishment in the dark, DET1 facilitates CRL4-COP1-SPA activity when promoting HY5 destabilization, although the molecular details of DET1 function in this regard are unknown (Osterlund et al., 2000). In addition, DET1 directly interacts and stabilizes basic helix-loop-helix Phytochrome interacting factors (PIFs) transcription factors, which trigger transcriptional responses under dark conditions, but act as transcriptional co-repressor in circadian rhythms (Lau et al., 2011; Dong et al., 2014). In the case of light-regulated seed germination, DET1, together with COP10, assembles into CRL4-DET1-COP10 complexes that bind PIF1, increasing its stability and activity in the dark to repress germination. Additionally, CRL4-DET1-COP10 target LONG HYPOCOTYL IN FAR-RED 1 (HFR1) protein for proteasomal degradation under dark conditions. HFR1 is a positive regulator of seed germination that forms heterodimers with PIF1, impeding PIF1 ability to bind to its target genes. By acting on different downstream effectors, DET1 becomes a central repressor of light-induced seed germination (Shi et al., 2015).

Recent work by Bourbousse et al., 2015, showed that both Arabidopsis DET1 and COP1 also regulate shaping of nuclear architecture in response to the light conditions. Thus, in etiolated cotyledons, DET1 and COP1 maintain heterochromatin in a decondensed state. Upon light exposure, cryptochrome-dependent light signalling releases nucleus expansion and large-scale heterochromatin condensation, likely by inhibiting COP1 and DET1 function. These global nuclear phenotypes correlate with RNA Polymerase II (RNAPII) transcriptional activity and may also impact gene silencing efficiency (Bourbousse et al., 2015). Although the molecular details of COP1 and DET1 function in these processes are still unknown, it is striking that both CRL4-associated proteins affect high order nuclear dynamics. Whether they participate in the same light-dependent regulatory mechanisms or in different additive ones needs to be unveiled.

Still regarding light perception, the UV RESISTANCE LOCUS 8 (UVR8) is also a β-propeller protein with seven blade-shaped β-sheets that acts as an UV-B photoreceptor in Arabidopsis (Rizzini et al., 2011). UVR8 has homology to human Regulator of Chromatin Condensation 1 (RCC1), which associates to chromatin by directly interacting with histones (Kliebenstein et al., 2002; Cloix and Jenkins, 2008). Though UVR8 physical association to chromatin is controversial (Binkert et al., 2016), it directly binds COP1 in a UV-dependent manner to protect HY5 against proteasomal degradation and to enhance *HY5* gene expression. Therefore, increased HY5 activity confers UV acclimation and protection to plants (Favory et al., 2009; Oravecz et al., 2006). Despite the fact that UVR8 does not contain a DWD motif, its activity is repressed by two redundant DWD proteins, REPRESSOR OF UV-B PHOTOMORPHOGENESIS 1 (RUP1) and RUP2. Thus, RUP1 and RUP2 induce photomorphogenesis by directly interacting with UVR8, mediating its dimerization and disrupting UVR8-COP1 interaction, which halts UV-B signalling (Gruber et al., 2010; Heijde and Ulm, 2013). Another DWD protein, DWD HYPERSENSITIVE TO UV-B 1 (DHU1), also contributes to repression of the UV-B signal transduction pathway by interfering with COP1 function. DHU1 likely works in association with RUP1 and is able to bind DDB1, suggesting a role as a substrate adapter for CRL4 complexes (Kim et al., 2017; Kim et al., 2014).

## DNA Lesion Detection and Repair – Light as a Damage

Plants use sunlight for photosynthesis and therefore cannot avoid constant exposure to solar UV radiation. To cope with the adverse effects of UV light, plants accumulate photoprotective pigments, such as flavonoids (i.e., anthocyanins) and carotenoids, and have evolved DNA damage repair mechanisms, such as photoreactivation and dark repair pathways (Stapleton and Walbot, 1994; Rozema et al., 2002; Kimura et al., 2004). In Arabidopsis, HY5 is a major regulator of anthocyanin accumulation in response to light (Ang and Deng, 1994). Therefore, production of the photoprotective pigment shield largely depends on the activity of COP1- and DET1-containing CRL4 E3 Ub ligases that promote HY5 destabilization (Holm et al., 2002). In the green algae *Chlamydomonas reinhardtii*, DET1 and DDB1 also repress photoprotective responses by regulating blue light-mediated induction of *LIGHT-HARVESTING COMPLEX STRESS-RELATED PROTEINS 1* and *3* genes, necessary for the dissipation of energy under high-light conditions (Aihara et al., 2019).

DNA repair mechanisms are essential for the survival of organisms since, if efficient repair of damaged DNA does not take place, genome integrity is at risk (Ries et al., 2000). In humans, unrepaired lesions might potentially introduce oncogenic mutations and are a major cause of skin carcinogenesis (Hoeijmakers, 2009). Among the lesions caused by UV light, cyclobutane pyrimidine dimers (CPDs) and pyrimidine (6–4) pyrimidinone dimers (6-4PPs) are the most common ones, accounting for 75% and 25% of DNA lesions, respectively, being able to stall RNAPII during transcription, which largely alters gene expression and cell functions (Mitchell et al., 1989; Fischer et al., 2011). Contrary to photoreactivation, dark repair pathways do not directly reverse DNA damage, but instead replace the damaged DNA with new, undamaged nucleotides. Dark repair mechanisms include the nucleotide excision repair (NER), base excision repair (BER), and mismatch repair (MMR) pathways (Scrima et al., 2011). DDB1 was initially identified as a damaged DNA binding protein that heterodimerizes with two DWD-containing proteins, Damage-specific DNA Binding protein 2 (DDB2) and Cockayne Syndrome A (CSA), that control different NER pathways, global genome repair (GGR) and transcription-coupled repair (TCR), respectively (Lee and Zhou, 2007; Shuck et al., 2008). Mutations in *CSA* and *DDB2* result in hereditary diseases, termed Xeroderma pigmentosum and Cockayne syndrome, characterized by cutaneous hypersensitivity to sunlight exposure and high susceptibility to UV-induced skin cancer, respectively (Lee and Zhou, 2007; Jackson and Xiong, 2009). Though they act through specific pathways, both CRL4-DDB2 and CRL4-CSA share common architectural features and a common mechanism of activation by CSN displacement from the CRL4-DCAF upon substrate binding to the DCAF (Fischer et al., 2011).

DDB2 and CSA function as part of CRL4 complexes that are conserved in plants (Biedermann and Hellmann, 2010). Thus, Arabidopsis *ddb2* mutants display enhanced sensitivity to UV irradiation, methyl methanesulfonate, and H_2_O_2_ stress (Koga et al., 2006) while overexpression of DDB1A increases plant tolerance to UV (Al Khateeb and Schroeder, 2009). Moreover, DDB2 associates with the CUL4-DDB1A complex to form an E3 ligase that modulates NER DNA damage repair (GGR associated) upon UV stress (Molinier et al., 2008). *CSAat1A*, a *CSA-like* gene, was identified in a forward screening for mutants with altered DNA damage responses. In Arabidopsis, there are two *CSA-like* genes that form heterotetramers within active CRL4 E3 ligases to enable plant responses to UV-B irradiation (Zhang et al., 2010).

The CDDD complex seems to be also essential for damage DNA repair processes in plants. Thus, it has been reported that an appropriate dosage of DET1 is necessary for efficient removal of UV photoproducts through the NER (GGR-type) pathway in Arabidopsis. In this context, DET1 is required for the CRL4-dependent degradation of DDB2 in order to complete the DNA repair process. Upon UV irradiation, DET1 is degraded concomitantly with DDB2, likely as a means to limit the activity of CUL4-DDB1-DET1-DDB2 complexes (Castells et al., 2011).

Similarly, DDA1 has been involved in the response to DNA damage in mammalian systems. Thus, *dda1* depleted human cells spontaneously accumulated double strand DNA breaks (Olma et al., 2009). Moreover, recent studies identify *DDA1* as an oncogene in various types of cancers and it is being considered as a therapeutic target (Cheng et al., 2017; Zhao et al., 2016; Gao et al., 2017). Recent structural data has shown that the 19 N-terminal amino acids of DDA1 adopt a partially coiled conformation that docks on a groove at the bottom surface of the DDB1 BPA domain. The authors hypothesized that the flexible C-terminal region of DDA1 might be able to reach DCAF or even DCAF-bound substrates to either facilitate recruitment of targets or modulate the overall topology of the fully assembled CRL4–target complex. These functions might be required for proper activation of CRL4 machineries during DNA damage responses (Shabek et al., 2018).

Despite the conservation of CRL4 machineries involved in NER in higher eukaryotes, photoreactivation, which is mediated by photolyases, is thought to be the major DNA repair pathway for CPDs and 6-4 PPs lesions in higher plants. Photolyases can specifically bind to these DNA lesions and remove them directly by absorbing energy (Molinier, 2017). Photoreactivation and photolyases have been reported in several plant species but are also common for many other organisms (Molinier, 2017; Kimura et al., 2004). Interestingly, Arabidopsis *det1* mutants display over-expression of DNA photolyase genes promoted by higher levels of HY5/HYH, resulting in increased sunscreen effect and greater tolerance to UV-C irradiation (Castells et al., 2010). Therefore, DET1, likely as part of CRL4-CDDD complexes, participates in different pathways leading to DNA protection and repair against UV light while being itself a repressor of light signalling. Whether regulatory relationships exist between DET1 functions in these two pathways should be addressed in future studies.

## Regulation of Histone and DNA Marks

Histone post-translational modifications (PTM) regulate chromatin accessibility and gene expression. A plethora of protein complexes tightly regulate the conjugation of activating or inactivating marks to histones facilitating or constraining the accessibility of transcription-complexes to specific genomic regions. Genetic and molecular evidence have shown that CRL4 play major roles in this process in Arabidopsis. Thus, CUL4-DDB1 complexes are known to interact with DWD protein MULTICOPY SUPPRESSOR OF IRA1 (MSI1), which is also an integral part of the evolutionary conserved POLYCOMB REPRESSIVE COMPLEX 2 (PRC2) that catalyses H3K27 trimethylation, a repressive histone mark (Dumbliauskas et al., 2011). Mutation of *CUL4* leads to autonomous endosperm development and loss of parental *MEDEA* imprinting, which is essential for seed formation, supporting a functional link between CRL4 and the PRC2 complex (Dumbliauskas et al., 2011). CRL4 also interacts with MSI4, which represses *FLC* expression through its association with PRC2. Thus, the lack of MSI4 or decreased CUL4 activity reduces H3K27 trimethylation on *FLC*, but also on its downstream target *FT*, resulting in increased expression of both genes during the regulation of flowering timing in Arabidopsis (Pazhouhandeh et al., 2011). In this context, it would be interesting to determine to which extent CRL4 function aids PRC2 activity and whether CRL4 ubiquitination activity is relevant for turnover of any PRC2 component as a means to modulate its gene repressor activity according to specific developmental or environmental conditions.

Non-DWD proteins that associate with DDB1 have been also shown to be key in integrating environmental cues to shape the Arabidopsis epigenome landscape, as recently shown for DET1 (Nassrallah et al., 2018). The idea that DET1 can act as a transcriptional repressor originally came with the finding that it has the capacity to directly interact with Histone 2B (H2B), more specifically with non-acetylated tails of H2B, which means that its interaction correlates with histone states compatible with transcriptional repression (Benvenuto et al., 2002). Recently, the significance of this interaction has been unveiled, since DET1 and the CDDD complex influence histone H2B monoubiquitination (H2Bub), a chromatin mark occurring over gene bodies that promotes light-dependent gene activation (Bourbousse et al., 2012; Nassrallah et al., 2018). Thus, it was found that the CDDD subunit DDA1 directly interacts with SAGA-INTERACTING FACTOR (SGF11), which, together with UBIQUITIN PROTEASE (UBP22) and ENHACER OF YELLOW2 (ENY2), comprise the Arabidopsis SAGA deubiquitination module (DUBm). Recognition of SGF11 by DDA1 recruits the CRL4-CDDD module to ubiquitinate and degrade the DUBm in a DET1- and dark- dependent manner. Therefore, Arabidopsis *det1* mutants display reduced levels of H2Bub mark as a consequence of the accumulation of DUBm and increased H2Bub deubiquitination activity (Nassrallah et al., 2018). Noticeably, *det1* mutants displayed altered accumulation of several other histone marks related with transcription activation, although the molecular mechanisms by which DET1 governs their accumulation and distribution over the genome are unknown (Nassrallah et al., 2018). In agreement with these findings, DET1 and COP1 had been previously shown to repress accumulation of the gene activating mark H3K9ac at specific loci. Thus, Arabidopsis *det1* and *cop1* mutants displayed increased H3K9ac levels in light regulated genes that correlated with their enhanced transcription levels. However the molecular basis underlying these phenotypes is still unknown (Guo et al., 2008).

Interestingly, Arabidopsis CDDD subunit COP10, which corresponds to an E2 Ub variant (i.e., lacks the cysteine residue at the catalytic centre required for Ub conjugation), has been shown to enhance the Ub binding activity of genuine E2 enzymes *in vitro*, including UBIQUITIN CONJUGATING ENZYME 1 (UBC1) and UBC2 (Yanagawa et al., 2004; Lau and Deng, 2009). The latter act in coordination with E3 Ub ligases HISTONE UBIQUITINASE 1 (HUB1) and HUB2 to ubiquitinate H2B (Xu et al., 2009). These findings suggest that COP10, and by extent the CRL4-CDDD, might modulate both H2B ubiquitination and deubiquitination.

H2Bub promotion of H3K4me3 deposition on actively transcribed genes is among the best studied trans-histone crosstalk in yeast and metazoans (Sun and Allis, 2002; Kim et al., 2009). In yeast, SET1 histone methyltransferase (HMT) catalyzes H3K4me3 deposition acting as part of the COMPASS (COMPlex of Proteins Associated with Set1), which also contains the WD40 repeat-containing proteins Swd1, Swd2, Swd3, among others (Schuettengruber et al., 2017). In Arabidopsis, the most relevant HMT in COMPASS is SDG2 (SET DOMAIN GROUP2). Recently, DWD-containing proteins SWD2-like a and b (S2La, S2Lb) were identified as the plant homologs of the Swd2 yeast protein. Together with SDG2, S2Lb directly influence H3K4me3 enrichment over highly transcribed genes, similar to its yeast ortholog. However, in Arabidopsis, H3K4me3 deposition seems not to rely in a trans-histone crosstalk with H2Bub (Fiorucci et al., 2019). In Arabidopsis, S2Lb was found to directly interacts with DDB1, indicating it might form part of CRL4 E3 Ub ligases (Beris et al., 2016). S2Lb also co-purifies with the AtCOMPASS core subunit WDR5a, also a WD40 protein. Interestingly, it has been shown that, in neuronal cells, CUL4B complexes can target WDR5 protein for degradation (Nakagawa and Xiong, 2011). Whether this mechanism is conserved in plants needs to be explored.

HIGH EXPRESSION OF OSMOTICALLY RESPONSIVE GENES 15 (HOS15), a DWD-repeat protein involved in cold tolerance, regulates the acetylation levels of cold responsive genes by targeting HISTONE DEACETYLASE 2C (HD2C) in a cold-dependent manner. This degradation is mediated by the CRL4 complex in which HOS15 acts as a substrate receptor (Park et al., 2018b). HOS15 can also directly interact with CBF transcription factors to modulate cold-induced binding to cold responsive gene promoters and promote the acquisition of cold tolerance (Zhu J. et al., 2008; Park et al., 2018b). Recently, it has been demonstrated that HOS15 is also a core component of the Arabidopsis HISTONE DEACETYLASE9-POWERDRESS (HDA9-PWR) complex (Suzuki et al., 2018; Mayer et al., 2019). *hos15* mutants display histone hyperacetylation similar to those of *hda9* and *pwr* mutants and ninety percent of HOS15-regulated genes are also controlled by HDA9 and PWR, being the majority of these genes stress-related (Mayer et al., 2019). In addition, HOS15 regulates HDA9 protein accumulation in the nucleus and its association to chromatin but does not affect the overall HDA9 protein stability (Mayer et al., 2019; Park et al., 2018a). Moreover, the HOS15-HDA9 module can directly interact with the Evening complex to reduce histone acetylation levels at the promoter of flowering activator *GIGANTEA* (*GI*). Therefore, the HOS15-HDA9 module could regulate photoperiodic flowering *via* transcriptional repression of *GI* (Park et al., 2019). Besides its interaction with HD2C, HDA9, and PWR, HOS15 was found to associate in affinity purification assays with other Histone deacetylases and Histones H1.2 variant, H2B and H4 (Park et al., 2018a; Zhu J. et al., 2008).

Altogether, these evidences indicate that DCAF proteins can affect histone posttranslational modification, either indirectly, by regulating the stability or activity of proteins or complexes that mediate deposition of histone marks, or directly, by acting as core components of such histone-modifying machineries.

Finally, recent studies suggest that the COP9 signalosome (CSN), a key regulator of CULLIN-based E3 Ub ligases, including CRL4, regulates DNA methylation in Arabidopsis (Tuller et al., 2018). The CSN consists of eight subunits (CSN1–8) and is highly conserved in eukaryotes. CSN was initially identified for its function as a repressor of photomorphogenesis. Indeed, *csn* display a *fusca* phenotype (characterized by high accumulation of anthocyanins, and small and unviable plants) and de-etiolation under dark conditions. Further characterization of the CSN uncovered its role in the regulation of CRL complexes recycling, by removing the Nedd8 peptide from the CRLs. Furthermore, characterization of the pleiotropic defects in *csn* mutants in Arabidopsis, Drosophila, and humans showed that the CSN has other functions besides the inhibition of the CRL E3 Ub ligase activity, that were related to its ability to associate with chromatin (Dessau et al., 2008; Singer et al., 2014; Chamovitz, 2009). Accordingly, a recent study suggested that the pleiotropic nature of the CSN is related to its function in regulating DNA methylation. Thus, Arabidopsis *csn8* and *csn5a-1* mutants showed similar methylation patterns, in overlapping positions, but rather differential to those of wild-type plants (Tuller et al., 2018). Moreover, this pattern correlated with changes in gene expression in these mutants. Although the mechanisms behind this regulation are still unknown, the authors suggested that this effect could be due to the impact of the CSN in the stability of many transcription factors, an interference with the activity of DNA methylation-related E3 ligases, or with the capacity of the CSN to directly bind DNA, as demonstrated in *Drosophila* (Kraft et al., 2008; Deng et al., 2016; Singer et al., 2014). In this context, the convergence of multiple regulatory mechanisms should not be excluded since CSN has an overall effect on diverse CRL4 E3 Ub ligases controlling chromatin accessibility, transcriptional regulation, and RNA stability and export. According to this notion, loss-of-function mutants for *CUL4* share similar pleiotropic phenotypes with *csn* mutants.

## mRNA Processing and Export

Mature mRNA formation requires the coordination of several processing mechanisms before export to the cytosol for translation occurs. A number of mRNA-binding proteins associates co-transcriptionally with the nascent mRNA to ensure its proper processing, including 5′ end capping, splicing and 3′ end polyadenylation, ending with the nuclear export of mature transcripts (Moore and Proudfoot, 2009).

Alternative polyadenylation has been only recently considered a widespread mechanism in regulating gene expression (Di Giammartino et al., 2011). The physiological relevance of this mechanism in plants can be illustrated by the function of DWD protein FY as a promoter of flowering transition. Arabidopsis FY is the homolog of the yeast and human RNA 3′ end processing factors, Pfs2p and WDR33, respectively, which associate to cleavage and polyadenylation complexes (Shi et al., 2009). FY is also able to directly interact with DDB1A/B and forms complexes with CUL4 (Lee et al., 2008). FY acts together with the nuclear RNA binding protein FCA within the flowering autonomous pathway to prevent accumulation of mature mRNAs of the floral repressor *FLC*. Indeed, both FY and FCA physically interact and both reduce RNA 3′ end processing of *FLC* transcripts, which downregulates *FLC* mRNA accumulation, allowing plant flowering (Simpson et al., 2003).

Splicing of precursor mRNAs (pre-mRNAs) through excision of noncoding regions (introns) and joining of adjacent coding regions (exons) is essential for the expression of nearly all eukaryotic protein-coding genes. Splicing is catalyzed by the spliceosome, a large and dynamic ribonucleoprotein (RNP) machinery located in the nucleus. Initially identified in *Caenorhabditis elegans*, Smu1 (suppressor of mec-8 and unc-52 1) is a spliceosome-associated WD40-repeat protein whose human ortholog interacts with CUL4B-DDB1 complexes *in vivo* (Higa et al., 2006; Spike et al., 2001). Although highly conserved in plants and metazoans, Smu1 is absent from budding yeast, suggesting that its function is required for complex splicing patterns (Ulrich et al., 2016). A recent study demonstrated that the Arabidopsis homolog SMU1 also acts as a splicing factor that influences splice site selection and alternative splicing patterns. SMU1 acts prior and during the first catalytic step of splicing and can be a general modulator of splicing patterns in plants (Kanno et al., 2017). It has been suggested that hSmu1, as well as Arabidopsis SMU1, could work as DCAF proteins in recognizing spliceosomal targets for ubiquitination (Higa et al., 2006; Chung et al., 2009). In agreement with this notion, key splicing factors undergo ubiquitination in Arabidopsis, suggesting a role for the ubiquitin-proteasome system in regulating spliceosome functions (Sasaki et al., 2015; Kanno et al., 2017). Whether SMU1 is involved in this regulatory mechanism in Arabidopsis remains to be investigated.

The recruitment of RNA export factors occurs in concomitance with transcription. For instance, the yeast THO/TREX (TRanscription-EXport) complex directly interacts with the C-terminal domain (CTD) of RNAPII during transcript elongation. This interaction enables THO/TREX complex recruitment to RNAPII-transcribed genes to facilitate transport of the nascent mRNAs (Meinel et al., 2013). In plants, our knowledge about this mechanism and the factors involved in coordinated mRNA biogenesis and export is much less known. Affinity purification of Arabidopsis TEX1/THO3-associated proteins showed that the THO core complex resembles that of metazoans, consisting of HPR1, THO2, THO5A/B, THO6, THO7A/B, and TEX1/THO3 (Yelina et al., 2010). TEX1/THO3 and THO6 are DWD proteins and THO6 forms a functional CRL4 E3 Ub ligase that plays also a role in ABA signalling (Lee et al., 2010). Mutants of *THO3/TEX1* and *THO6* produce reduced amounts of small interfering siRNA, suggesting an additional role of the Arabidopsis THO/TREX in siRNA biosynthesis (Yelina et al., 2010; Jauvion et al., 2010; Sørensen et al., 2017).

The nucleoporin RAE1 (RNA Export Factor 1) is part of the nuclear pore complex (NPC) that acts as a shuttling transport factor at the nuclear envelope to allow mRNAs export (Pritchard et al., 1999; Okamura et al., 2015). Transport between the nucleoplasm and the cytoplasm depends on mRNA recognition by the NPC, which is the largest multiprotein complex in eukaryotic cells. Mass spectrometry analysis of GFP-RAE1 interactors suggested that the Arabidopsis NPC protein composition is very similar to that of vertebrates (Tamura et al., 2010). Arabidopsis RAE1 is itself a DWD domain-containing protein that interacts with DDB1A in Y2H assays (Lee et al., 2008). It would be interesting to determine if RAE1 is a canonical DCAF and to identify its protein targets, which could include other NPC components, chaperones, and, very importantly, passenger proteins or RNAs.

## miRNA Biogenesis

Among the known miRNA biogenesis factors in Arabidopsis, the DWD containing proteins PLEIOTROPIC REGULATORY LOCUS1 (PRL1), PRL2, and MAC3B belong to the MOS4-associated complex (MAC) (Lee et al., 2008; Monaghan et al., 2009). MAC is a highly conserved complex among eukaryotes, with its yeast and humans orthologs known as the NineTeen Complex (NTC) and Prp19 complex (Prp19C), respectively. The Arabidopsis MAC, as the yeast and human NTC/Prp19C, associates with the spliceosome. All three complexes are predicted to share conserved functions in regulating splicing (Monaghan et al., 2009; Johnson et al., 2011). In Arabidopsis, loss-of-function of MAC, as in *mac3a mac3b* and *prl1 prl2* double mutants, impairs miRNA biogenesis (Jia et al., 2017; Li et al., 2018). Both MAC3B and PRL1 were shown to have E3 Ub ligase activity *in vitro*. In fact, it has been reported that PRL1 likely acts as a substrate receptor involved in the degradation of the AKIN10 kinase, a key regulator of cell energy homeostasis (Lee et al., 2008; Wiborg et al., 2008; Monaghan et al., 2009).

## Ribosome Biogenesis

Ribosomes are fundamental macromolecular machines and the basis of the translation machinery, allowing the conversion of information encoded within mRNAs into proteins. The 80S ribosome is a ribonucleoprotein complex that comprises two ribosomal subunits, a large 60S subunit (containing the 25S, 5.8S, and 5S rRNAs, and 46 ribosomal proteins) and a small 40S subunit (containing the 18S rRNA and 33 ribosomal proteins) (Fromont-Racine et al., 2003; Henras et al., 2008). Ribosome biogenesis is an essential process for cell growth and proliferation. Though it is best characterized in yeast by genetic and proteomic studies (Henras et al., 2008; Panse and Johnson, 2010), plants have homologs of yeast and mammalian ribosomal biogenesis factors, despite only a few of them have been studied (Pendle et al., 2005; Horiguchi et al., 2011). Interestingly, a number of DWD proteins have been associated with ribosome biogenesis. For instance, SLOW WALKER1 (SWA1) is a DWD with six WD40 repeats involved in 18S rRNA processing (Shi et al., 2005). Characterization of the semisterile Arabidopsis mutant *swa1* showed it is defective in mitotic progression of the female gametophyte, pointing to an essential role for SWA1 during megagametogenesis (Shi et al., 2005). Gametophyte and embryo sac development requires another DWD protein, PERIODIC TRYPTOPHAN PROTEIN 2 (PWP2), a homolog of the yeast Pwp2 protein involved in nucleolar processing of pre-18S ribosomal RNA for 40S subunit maturation (Missbach et al., 2013). Despite the presence of putative DWD domains, it is unknown whether SWA1 and PWP2 can associate with CRL4 complexes and form functional E3 ligases.

Arabidopsis YAOZHE (YAO) and EMB2271 are related DWD proteins that display homology to the non-ribosome nucleolar protein Rrp9/hU3-55K from yeast and humans, respectively. Rrp9/hU3-55K are present in the 90S pre-ribosome, being essential for 18S rRNA maturation and 40S subunit biogenesis (Grandi et al., 2002). Mutation of *YAO* in Arabidopsis reduces competence of male gametophytes as well as, in a reduced number of cases, impairs development of the embryo sacs at the four-nucleate stage, showing aberrant nuclear positioning (Li et al., 2010). In addition, *yao* mutants display misplacement of the cell plate in the zygote and subsequent zygote arrest, leading to early embryo lethality. Thus, YAO is required for gametophyte development and regulation of cell division planes during embryogenesis (Li et al., 2010). Though a direct interaction with CRL4-DDB1 machinery would be expected, Li and co-workers could not detect physical interaction between YAO and DDB1A or DDB1B using yeast two-hybrid assays (Li et al., 2010).

The maturation and assembly of the 60S ribosomal subunit is also regulated by DWD domain proteins. PEP2 (PESCADILLO ORTHOLOG 2) is the Arabidopsis homolog of human DWD protein WDR12, which is involved in the maturation of 28S and 5.8S subunits during the formation of the 60S ribosome (Zografidis et al., 2014). Arabidopsis PEP2 directly interacts with PESCADILLO (PES), whose homolog in zebrafish is essential for embryonic development. In Arabidopsis PES, BOP (Block of Proliferation 1) and WDR12 also constitute the evolutionarily conserved PeBoW complex involved in cell growth and differentiation that is indispensable for viability of yeast and higher eukaryotes. In response to nucleolar stress or DNA damage, Arabidopsis PeBoW proteins move from the nucleolus to the nucleoplasm. Depletion of PeBoW proteins led to dramatic suppression of cell proliferation, expansion, and differentiation. Concomitantly, PeBoW silencing caused rapid transcriptional modulation of cell-cycle genes, suggesting that, by affecting ribosome biogenesis, this complex plays a critical role in plant cell growth and survival (Ahn et al., 2016). Another DWD protein NOTCHLESS (NLE) is involved in maturation and assembly of the 60S ribosomal subunit, and is essential for proper cellular growth and proliferation during plant development (Chantha and Matton, 2007). Silencing of the *NLE* gene by RNA interference in Arabidopsis led to a semisterile phenotype caused by defects in female gametophyte development, further supporting a role for ribosome biogenesis in female gametophyte development in plants (Chantha et al., 2010).

## General Conclusions and Future Perspectives

Due to their three dimensional structural features, DWD proteins are prone to be involved in protein–protein interactions. Indeed, numerous DWD proteins are components of different multi-subunit complexes including CRL4 E3 Ub ligases. However, a large number of Arabidopsis DWD proteins referred here have not been confirmed as physical interactors of DDB1. It is also unknown whether they contribute to CRL4 E3 Ub ligase activities and which are their precise targets. These might include either unrelated protein substrates (i.e., transcriptional regulators, metabolic enzymes, and structural proteins) or even components of the multi-subunit complexes to which they belong, being necessary to regulate protein complex assembly and/or function. Nevertheless, it cannot be excluded that many of these proteins have conserved the DWD domain during evolution but have lost the capacity to interact with CRL4 complexes, gaining new abilities in the meanwhile.

To date, at least 53 Arabidopsis DWD proteins have been partially characterized. Among those, 32 display a function related to transcriptional output either by regulating abundance of transcription factors, by modulating chromatin accessibility or transcript processing. This represents a paradox since, by exerting a tight and selective control of protein degradation, CRL4-associated proteins can regulate transcriptional events and assure correct protein synthesis. Importantly, CRL4 function on nuclear events is very likely not limited to targeted degradation of chromatin-associated factors. Indeed, it is known that CRL4 complexes mediate different kinds of non-proteolytic ubiquitination of protein substrates, including formation of K63-linked polyUb chains and monoUb conjugates (Sugasawa et al., 2005). Such CRL4-mediated modifications, instead of triggering degradation of targets, may influence their protein–protein interaction ability, DNA binding capability, or their subcellular localization (Deshaies and Joazeiro, 2009; Dumbliauskas et al., 2011).

The idea that CRL4 ligases are genome caretakers due to their prominent role during DNA replication and repair is not new (Lampert et al., 2017). In Arabidopsis, similar to the case of other organisms, this surveying function can be extended to other mechanisms directly related with DNA transcription. Such a remarkable pleiotropy of functions can only be achieved by the versatility and modular function of CRL4 complexes that assemble into a plethora of functional E3 ligases just by exchanging the substrate recognition modules. It is therefore not surprising that DDB1 was scored as an essential gene in a human genome-wide CRISPR/Cas9 knockout screen (Hart et al., 2015). This situation is analogous to the case of Arabidopsis where total loss of DDB1 function (as shown in null *ddb1a ddb1b* double mutants) causes embryolethality (Bernhardt et al., 2010).

Despite our increasing knowledge on the chromatin-associated functions of Arabidopsis DCAFs, a large number of them are still orphan substrate receptors, whose function is completely unknown. Further efforts should be dedicated to fill this gap, providing a better understanding on the roles of CRL4-associated DCAFs in shaping nuclear architecture, epigenetic landscape, and gene expression throughout the plant life cycle.

## Author Contributions

SF gathered information, designed and wrote the manuscript, and drew **Figure 1**.

VR revised and edited the manuscript.

## Funding

SF has a Ramon y Cajal grant RYC-2014-16308 funded by the Ministerio de Economia y Competitividad. Work in VR laboratory is funded by the Project BIO2016-80551-R by Agencia Estatal de Investigación/Fondo Europeo de Desarollo Regional/European Union.

## Conflict of Interest Statement

The authors declare that the research was conducted in the absence of any commercial or financial relationships that could be construed as a potential conflict of interest.
